# A Novel Loss-of-Function Variant in *TGFBI* Associated with Non-Syndromic Orofacial Clefts in a Chinese Pedigree

**DOI:** 10.3390/genes17090999

**Published:** 2026-08-25

**Authors:** Shujie Hou, Siyao Li, Xinluo Wang, Shiying Zhang, Yuntao Lu, Ting Zhang, Zhibo Zhou, Wenbin Huang, Xuedong Wang, Bing Han, Jieni Zhang

**Affiliations:** 1 Department of Orthodontics, Peking University School and Hospital of Stomatology, National Center for Stomatology Beijing/National Clinical Research Center for Oral Diseases Beijing/National Engineering Research Center of Oral Biomaterials and Digital Medical Devices Beijing, Beijing 100081, China; 2 Department of Oral and Maxillofacial Surgery, Peking University School and Hospital of Stomatology, National Center for Stomatology Beijing/National Clinical Research Center for Oral Diseases Beijing/National Engineering Research Center of Oral Biomaterials and Digital Medical Devices Beijing, Beijing 100081, China; 3 Guangdong Provincial High-Level Clinical Key Specialty, Guangdong Province Engineering Research Center of Oral Disease Diagnosis and Treatment, Department of Orthodontics, Stomatological Center, Peking University Shenzhen Hospital, Shenzhen Peking University-The Hong Kong University of Science and Technology Medical Center, Shenzhen 518036, China; 4 Center of Stomatology, Beijing Tsinghua Changgung Hospital, School of Clinical Medicine, Tsinghua Medicine, Tsinghua University, Beijing 102218, China; wang-xuedong@tsinghua.edu.cn; 5 Institute of Advanced Clinical Medicine, Peking University, 38 Xueyuan Road, Haidian District, Beijing 100191, China

**Keywords:** non-syndromic orofacial cleft, craniofacial anomaly, whole-exome sequencing, *TGFBI*, loss-of-function mutation

## Abstract

**Background/Objectives**: Non-syndromic orofacial clefts (NSOFCs) are among the most common congenital craniofacial anomalies, with genetic factors playing a major role in their etiology. Transforming growth factor-β-induced (TGFBI) protein is an extracellular matrix protein implicated in cell adhesion and apoptosis. Although experimental studies have implicated TGFBI in palatal fusion, its contribution to human NSOFCs remains unclear. This study investigated the genetic basis of hereditary NSOFCs in a Chinese family. The functional consequences of the identified *TGFBI* variant were subsequently evaluated. **Methods**: Whole-exome sequencing was performed in a Chinese NSOFC pedigree, followed by Sanger validation and ophthalmological evaluation. The functional impact of the identified variant was investigated through evolutionary and structural analyses, TGFBI expression analysis during mouse palatal development, and cellular functional assays. **Results**: A novel heterozygous stop-gain variant in *TGFBI* (NM_000358.3:c.230C > A; p.Ser77X) was identified in affected family members but not in the unaffected father. No corneal abnormalities were observed in variant carriers. TGFBI was expressed in the midline epithelial seam during palatal fusion. The p.Ser77X variant generated a truncated protein lacking all four FAS1 domains and the C-terminal RGD motif, resulting in abnormal localization, impaired cellular apoptosis and proliferation, and altered p38-MAPK signaling. **Conclusions**: This study identifies a rare loss-of-function *TGFBI* variant associated with NSOFCs and provides genetic and functional evidence supporting a role for *TGFBI* variation in human palatal development.

## 1. Introduction

Nonsyndromic orofacial clefts (NSOFCs), which include cleft lip (CL), cleft lip with palate (CLP), and cleft palate (CP), are among the most common congenital craniofacial anomalies, affecting approximately 1 in 700 live births worldwide [1,2]. These defects arise from complex interactions between genetic susceptibility and environmental factors [3,4]. Orofacial clefts and tooth agenesis also share several genetic and developmental pathways, suggesting partially overlapping mechanisms during craniofacial development [5]. To date, genome-wide association studies (GWASs) have proven instrumental in mapping common susceptibility loci for NSOFCs, including *IRF6* [6] and *GRHL3* [7]. These variants collectively explain only a modest fraction of disease heritability. Notably, a significant proportion of GWAS signals are located within non-coding regulatory elements, posing a substantial challenge to the identification of causal genes and the elucidation of pathogenic mechanisms. Beyond common variants, rare variants identified through targeted sequencing and whole-exome sequencing (WES) have emerged as important contributors to NSOFCs pathogenesis [8].

During palatogenesis, the medial edge epithelium (MEE) at the tips of the palatal shelves mediates adhesion and forms the midline epithelial seam (MES). A transient periderm layer overlies the MEE. Disruption of the regulatory network impairs periderm differentiation and clearance, which in turn results in periderm persistence, failed seam resolution, and ultimately cleft palate [9].

Transforming Growth Factor Beta-Induced (TGFBI) protein, originally termed βig-H3, is a secreted extracellular matrix protein of 683 amino acids whose expression is induced by TGF-β signaling. Structurally, it consists of an N-terminal signal peptide, a cysteine-rich CROPT domain (previously referred to as the EMI domain), four tandem Fas1 (FAS1) domains, and a C-terminal Arg-Gly-Asp (RGD) sequence [10]. TGFBI functions as a matricellular adapter, modulating cell adhesion, migration, and proliferation through interactions with integrins [11]. It is listed in OMIM (MIM: 601692) and is associated with several autosomal dominant corneal dystrophies. In addition, functional inhibition of *Tgfbi* in mouse embryonic models has been reported to result in failed MEE apoptosis and a high frequency of secondary cleft palate [12]. However, to date, no direct clinical evidence has confirmed that *TGFBI* mutations cause this phenotype in human patients.

In this study, we performed WES in a family with NSOFCs and identified a rare heterozygous stop-gain variant in *TGFBI*. Bioinformatic prediction and functional validation in HEK293T cells revealed that this variant results in severe protein truncation and loss of normal protein function. Our findings identified a novel loss-of-function variant in *TGFBI* associated with human NSOFCs, providing insights into the possible role of this gene in palate development.

## 2. Materials and Methods

### 2.1. Ethical Compliance

This investigation was conducted in accordance with protocols approved by the Biomedical Ethics Committee of Peking University School and Hospital of Stomatology (approval code: PKUSSIRB-2025116203; approval date: 27 November 2025). Written informed consent was secured from all participants and/or their legal guardians. All mouse experiments of this study were approved by the Committee on the Ethics of Animal Experiments of Peking University School and Hospital of Stomatology (approval code: BDKQ-202505300526; approval date: 30 May 2025) and performed in strict accordance with the animal care and use guidelines.

### 2.2. Patients and Whole-Exome Sequencing

Demographic and clinical data—including age, sex, medical history, and pedigree information—were systematically documented. We clinically identified a nonsyndromic cleft lip and palate proband, and history-taking suggested that the proband’s mother and some distant relatives might have similar conditions. For further genomic analysis, blood samples were collected from the proband, his family members, and unrelated healthy controls. Peripheral blood lymphocytes served as the source for genomic DNA extraction, which was performed using the TIANamp Genomic DNA Kit (DP304, TIANGEN, Beijing, China) according to the manufacturer’s protocol. All family members underwent comprehensive ophthalmic evaluations at a local clinic, including routine visual acuity testing, slit-lamp biomicroscopy, corneal topography, and anterior segment photography. The affected family members reported no history of other congenital abnormalities or cardiovascular, renal, or urinary tract disorders.

Whole-exome sequencing was performed on genomic DNA from the proband, the affected mother, and the unaffected father using the Illumina NovaSeq platform with 150 bp paired-end reads. The mean sequencing depth on target regions was 134×, with 99.69% of target bases covered at ≥10×. Raw reads were aligned to the human reference genome (GRCh37/hg19) using BWA-MEM (v0.7.17), and duplicate reads were marked using Sambamba (v0.6.8).

Variant calling was performed using bcftools (v1.16). Variant annotation was conducted with ANNOVAR (2023Feb1), incorporating population frequency databases (1000 Genomes, ESP6500, gnomAD v2.1.1), functional prediction tools (SIFT, Polyphen-2, MutationTaster, CADD, GERP++, dbscSNV), and disease association databases (ClinVar, OMIM, HGMD). ACMG classification was performed according to the 2015 ACMG/AMP guidelines.

Candidate pathogenic variants were identified through a stepwise filtering strategy:

The vcf files of SNPs and INDELs from the proband, affected mother, and unaffected father were merged and annotated. The deleteriousness of the mutant loci was prioritized by filtering with different filtering conditions. The filtering conditions were as follows:

(1) Variants with a frequency higher than 1% in any of the 1000 Genomes data (1000g_all), ESP6500 data (esp6500siv2_all), and gnomAD data (gnomAD_ALL and gnomAD_EAS) were excluded. After this step, the initial set of 189,580 SNVs and small indels was reduced to 7373 variants.

(2) Only variants located in exonic regions or splicing regions were retained, leaving 1410 variants.

(3) Synonymous SNP mutations that were not located in highly conserved regions and were not judged by prediction software to affect splicing were filtered out. Small (<10 bp) non-frameshift InDel mutations in repetitive regions were also filtered out, resulting in 876 variants.

(4) Mutations evaluated by at least half of the four software programs (SIFT, Polyphen, MutationTaster, and CADD) [13] with available scores as potentially affecting protein function were retained. InDel mutations with no predictive scores were retained. SNP mutations predicted by dbscSNV to affect splicing were retained. Variants with splicing site mutations located within ±2 bp of exon boundaries were also retained. This step yielded 601 candidate variants.

(5) Inheritance model filtering: SNP and InDel loci that passed deleteriousness filtering were subjected to inheritance model screening using in-house software. For dominant inheritance screening, loci were retained where the patients were heterozygous and the unaffected individual was homozygous for the reference allele. For recessive inheritance screening, two models were assessed: (i) homozygous recessive model—loci where the proband was homozygous for the alternate allele and parents were heterozygous or reference; (ii) compound heterozygous model—loci where a gene harbored at least two heterozygous variants in the proband, with neither parent homozygous for the alternate allele, and the proband’s variant distribution could not be identical to, nor a subset of, that of either parent. After dominant inheritance screening, 143 candidate genes were identified. No variants consistent with recessive or compound heterozygous inheritance were identified (Table S1).

(6) ACMG classification filtering: To further prioritize variants with the strongest pathogenic potential, we intersected the 143 candidate genes with variants classified as “Pathogenic” or “Likely Pathogenic” according to the ACMG guidelines (n = 37 in the filtered dataset). This intersection yielded 28 candidate variants (Table S2).

(7) Among the 28 candidate genes, we retained variants of the following types: frameshift deletions (1), frameshift insertions (1), startloss (1), and stopgain (1), while filtering out missense SNVs (24). After filtering, four high-confidence candidate variants remained. Our literature review led us to prioritize *TGFBI* c.230C > A (p.S77X) for further investigation (Table A1).

To explore whether additional rare *TGFBI* variants were present in unrelated cases, WES data from an independent cohort of sporadic NSOFC patients were screened for rare coding variants in *TGFBI*. Candidate variants were evaluated according to population frequency and in silico predictions and were subsequently validated by Sanger sequencing.

### 2.3. PCR–Sanger Sequencing

PCR amplification followed by Sanger sequencing was carried out using site-specific primers (forward: 5′-ATCTTCCTAATTCTGCTGCT-3′; reverse: 5′-TCCTAAAAATGCCACTCCCT-3′) targeting the chr5:135369547 locus and with primers (forward: 5′-GAGGTTATCGTGGAGTGGAC-3′; reverse: 5′-GCCTACCTGAGTCTGGGATG-3′) targeting the chr5:135388680 locus.

### 2.4. Bioinformatic Analysis

The amino acid sequence of wild-type human TGFBI (NP_000349.1; 683 amino acids) was retrieved from the NCBI Protein database. Based on the p.S77X stop-gain variant, a truncated sequence comprising residues 1–76 was generated. The wild-type and truncated sequences were submitted separately to the AlphaFold3 [14] Server (https://alphafoldserver.com/, accessed on 25 October 2025), and the top-ranked prediction (Model 0) for each sequence was selected. The predicted structures were visualized and superimposed using PyMOL (v3.1.0) (Schrödinger, LLC., New York, NY, USA). Following structural superposition of the mutant model with the corresponding N-terminal region of wild-type TGFBI, an RMSD of 3.267 Å was obtained across 52 aligned atom pairs. The susceptibility of the *TGFBI* c.230C > A (p.S77X) variant to nonsense-mediated mRNA decay (NMD) was evaluated using NMDEscPredictor (https://nmdprediction.shinyapps.io/nmdescpredictor/, accessed on 16 August 2026) based on the reference transcript NM_000358.3.

### 2.5. Plasmid Construction

Empty vector (CV015) and wild-type *TGFBI* plasmids (HG10569-CH) were obtained from SinoBiological (Beijing, China), and p.S77X mutant *TGFBI* was constructed by SinoBiological (Beijing, China). Each of the constructed plasmids was subjected to Sanger sequencing for sequence verification.

### 2.6. Bulk RNA Sequencing (RNA-Seq) Analysis

#### 2.6.1. RNA Extraction

Cell lysates were submitted to Shanghai Majorbio Biopharm Technology Co., Ltd. (Shanghai, China) for RNA extraction using TRIzol^®^ Reagent following the manufacturer’s protocol. RNA integrity and concentration were subsequently assessed with a 5300 Bioanalyzer (Agilent, Hong Kong, China) and quantified using a NanoDrop 2000 spectrophotometer (NanoDrop Technologies, Waltham, MA, USA). Only specimens meeting the esteblished quality criteria—OD260/280 ratio between 1.8 and 2.2, OD260/230 ratio ≥ 2.0, RNA Quality Number (RQN) ≥ 6.5, 28S:18S rRNA ratio ≥ 1.0, and total RNA yield > 1 μg—were used for subsequent library construction, which was also performed at Shanghai Majorbio Biopharm Technology Co., Ltd., Shanghai, China.

#### 2.6.2. Library Preparation and Sequencing

Library construction and sequencing were carried out in accordance with the manufacturer’s protocols (Illumina, San Diego, CA, USA). Starting with 1 μg of total RNA, the RNA-seq transcriptome library was prepared using the Illumina^®^ Stranded mRNA Prep Ligation kit. Briefly, polyadenylated mRNA was isolated via oligo(dT) bead-based selection and subsequently fragmented using fragmentation buffer. First-strand cDNA synthesis was performed with random hexamer primers (Illumina), followed by second-strand synthesis using the SuperScript double-stranded cDNA synthesis kit (Invitrogen, Carlsbad, CA, USA). The resulting double-stranded cDNA underwent end repair, phosphorylation, and A-tailing as specified in the Illumina library construction protocol. Fragments of approximately 300 bp were size-selected on 2% Low-Range Ultra Agarose and then amplified by PCR for 15 cycles using Phusion DNA polymerase (NEB). After quantification with a Qubit 4.0 fluorometer, the prepared library was sequenced on the NovaSeq X Plus platform with paired-end 150 bp reads.

#### 2.6.3. Quality Control and Read Mapping

Raw paired-end reads were first processed using fastp [15] with default parameters for trimming and quality control. The resulting clean reads were then aligned to the reference genome in orientation mode using HISAT2 [16]. For each sample, transcript assembly was performed with StringTie [17] employing a reference-based approach. All subsequent data analyses were conducted on the Majorbio Cloud platform (www.majorbio.com).

#### 2.6.4. Differential Expression Analysis and Functional Enrichment

Transcript expression levels were quantified using the transcripts per million reads (TPM) method, with gene abundance estimation performed by RSEM [18]. To identify differentially expressed genes (DEGs) between sample groups, analysis was conducted using either DESeq2 [19] or DEGseq [20]. Genes meeting the criteria of |log2 fold change| ≥1 and a false discovery rate (FDR) ≤ 0.05 (for DESeq2) or FDR ≤ 0.001 (for DEGseq) were considered significantly differentially expressed. Subsequently, functional enrichment analysis was performed to identify overrepresented Gene Ontology (GO) terms and Kyoto Encyclopedia of Genes and Genomes (KEGG) pathways among the DEGs, applying a Bonferroni-corrected *p*-value ≤ 0.05 as the significance threshold relative to the whole-transcriptome background. GO enrichment was assessed using Goatools, and KEGG pathway analysis was conducted with KOBAS [21,22].

### 2.7. Materials and Antibodies

RIPA lysis buffer (P0013B) was obtained from Beyotime (Shanghai, China). Penicillin–streptomycin (P1400) and protein ladder (PR1920) were obtained from Solarbio (Beijing, China). The BCA protein assay kit (PA115) was obtained from TIANGEN (Beijing, China). DMEM (C11995500), trypsin-EDTA (25200056), and fetal bovine serum (FBS) (A5256701) were obtained from Gibco (Grand Island, NY, USA). 6 × His, His-Tag Monoclonal antibody (Cat No. 66005-1-Ig), β-actin antibody (Cat No. 66009-1-Ig), and HRP-conjugated secondary antibody (anti-rabbit and anti-mouse) (Cat No. SA00001-7H, Cat No. SA00001-1-A) were obtained from Proteintech (Wuhan, China); caspase-3 antibody (ab32150), cleaved caspase-3 antibody (ab32042), and TGFBI antibody (Ab169771) were obtained from Abcam (Cambridge, MA, USA); and p38 MAPK antibody (8690T), phospho-p38 MAPK antibody (4511T), SMAD2 XP antibody (5338T), and Phospho-SMAD2 antibody (18338T) were obtained from Cell Signaling Technology (Danvers, MA, USA).

### 2.8. Cell Culture and Transfection

HEK293T cells were cultured in DMEM (Gibco, Grand Island, NY, USA) supplemented with 10% fetal bovine serum (FBS) (Gibco, Grand Island, NY, USA) and 1% penicillin–streptomycin (Solarbio, Beijing, China) at 37 °C in a 5% CO_2_ humidified atmosphere. Cells were harvested and seeded in six-well plates at a density of 1 × 106 cells per well. When the confluence reached 70%, cells were transfected with an empty vector or with wild-type or p.S77X mutant *TGFBI* plasmids using Lipofectamine™ LTX Reagent with PLUS™ Reagent (Thermo Fisher Scientific, Waltham, MA, USA) following the manufacturer’s protocol. Cells were harvested 48 h after transfection for subsequent analyses. The empty vector served as the control.

### 2.9. Immunofluorescence

Immunofluorescence was utilized to determine the localization of the TGFBI protein in tissues and cells. To detect endogenous expression in the palate, embryonic day 13.5 and 14.5 C57BL6/J mouse embryos were collected, fixed in 4% paraformaldehyde, dehydrated, embedded in paraffin, and sectioned at about 4 µm. After deparaffinizing in xylene and rehydrating, sections were retrieved for heat-induced antigen in EDTA buffer (pH 9.0). After cooling to room temperature (RT), sections were washed in PBS, permeabilized with 0.1% Triton X-100, blocked and then incubated with anti-TGFBI primary antibody (Dilution 1:250) at 4 °C overnight. After washing the excessive primary antibody with PBS three times, the samples were incubated with fluorescent secondary antibody (Dilution 1:5000). Finally, the nucleus was counterstained with DAPI. For cell culture, transfected HEK293T cells were fixed with 4% paraformaldehyde for 10 min and permeabilized with 0.3% Triton X-100 for 15 min. Then, cells were incubated with anti-His tag primary antibody (Dilution 1:2000) overnight at 4 °C, followed by incubation with a fluorophore-conjugated secondary antibody (Dilution 1:2000). Nuclei were stained with DAPI. The images were captured under a confocal microscope (STELLARIS 8, Leica).

### 2.10. Western Blot

Total protein of transfected HEK293T cells was extracted using RIPA lysis buffer (Beyotime, Shanghai, China) containing protease and phosphatase inhibitors. Protein concentrations were determined using a BCA protein assay kit (TIANGEN, Beijing, China) according to the manufacturer’s instructions. Lysates were separated by SDS-PAGE and transferred to PVDF membranes. Membranes were blocked with 5% BSA solution at room temperature (RT) and incubated with primary antibodies against His-tag (Dilution 1:10,000), caspase-3 (Dilution 1:5000), cleaved caspase-3 (Dilution 1:500), p38 (Dilution 1:1000), p-p38 (Dilution 1:1000), and β-actin (Dilution 1:10,000) overnight at 4 °C. After washing 3 times with TBST, membranes were incubated with HRP-conjugated secondary antibodies (Dilution 1:10,000) at RT for 2 h. Finally, protein bands were visualized using a chemiluminescent kit (Millipore, St. Louis, MO, USA) and an imaging system (eBLOT Touch Imager™, Shanghai, China). Quantification analysis was performed using ImageJ (v1.54p) (NIH, Bethesda, MD, USA) while β-actin was used as the internal loading control.

### 2.11. Cell Apoptosis Assay

The effect of the p.S77X mutation on TGFBI-induced apoptosis was evaluated with an Annexin V-FITC/PI Apoptosis Detection Kit (AD11, DOJINDO, Kumamoto, Japan) following the supplier’s protocol. Cells transfected with the appropriate plasmids were collected 48 h after transfection, washed twice with cold PBS, and resuspended in 1× binding buffer to a final concentration of 1 × 10^6^ cells/mL. Aliquots of 100 μL from each suspension were incubated with 5 μL of Annexin V-FITC and 5 μL of PI for 15 min at room temperature in the dark, after which 400 μL of binding buffer was added. Flow cytometric analysis was performed within 1 h on a Beckman Coulter instrument, with at least 10,000 events recorded per sample using FITC and PI channels. Apoptotic rates were determined by summing the percentages of early apoptotic (Annexin V^+^/PI^−^) and late apoptotic/necrotic (Annexin V^+^/PI^+^) cells. Data were processed and visualized with Flowjo V10 software, utilizing unstained and single-stained samples for compensation and gating.

### 2.12. Cell Proliferation Assay

For the EdU incorporation assay, a 10 min incubation with EdU (5-ethynyl-2′-deoxyuridine) from the assay kit (Beyotime, Shanghai, China) was done. Then transfected cells were first fixed in 4% paraformaldehyde and permeabilized with 0.1% Triton X-100. The cells were mounted with DAPI-containing antifade medium (Beyotime, Shanghai, China). Imaging was performed on a STELLARIS 8 confocal microscope (Leica), and the percentage of EdU-positive cells was quantified using ImageJ software (NIH, Bethesda, MD, USA). All procedures were conducted in accordance with the manufacturer’s instructions.

### 2.13. Statistical Analysis

Data are expressed as the mean ± standard deviation (SD). One-way analysis of variance (ANOVA) was utilized to assess statistical significance across multiple groups, and when the overall test indicated significance, pairwise comparisons were carried out using Tukey’s multiple-comparisons test. A *p*-value < 0.05 was considered statistically significant. Analysis was performed using GraphPad Prism 10.1.2.

## 3. Results

### 3.1. Identification of a Novel Heterozygous Truncating Variant in TGFBI in a Familial NSOFC Case

**Figure 1:**
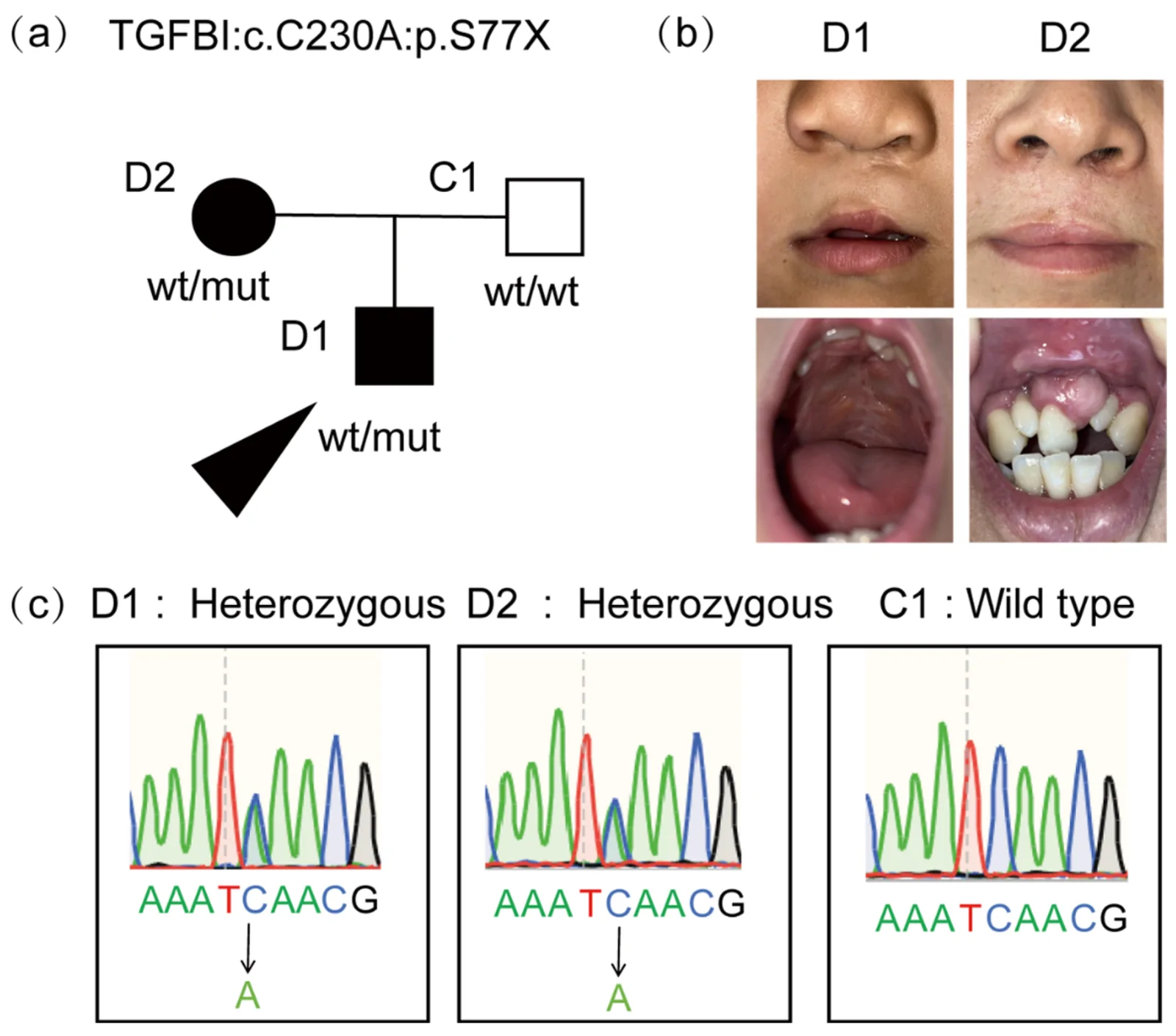
Identification of the heterozygous variant of *TGFBI* in a family with NSOFCs. (**a**) Pedigree of the family with NSOFCs. The arrow indicates the proband (D1). Filled symbols represent affected individuals (D1 and D2) with NSOFCs. (**b**) Clinical photographs of the lip, palate, and alveolus from subjects D1 and D2; (**c**) Sanger sequencing chromatograms confirming the heterozygous c.230C > A (p.S77X) variant in *TGFBI*. Arrows indicate the mutated loci.

**Figure A1:**
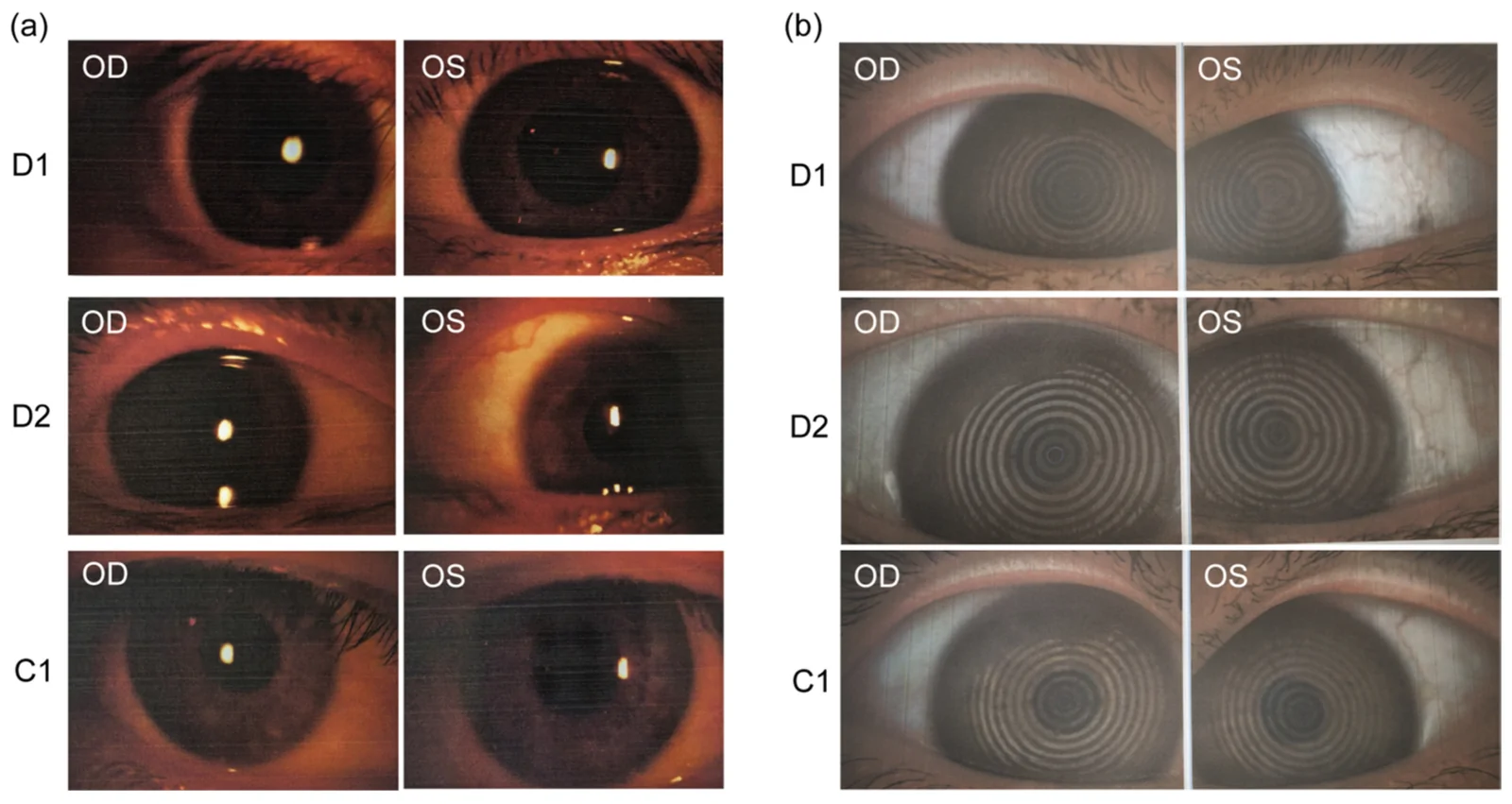
Anterior segment photography (**a**) and corneal topography (**b**) of the family members. D1, proband; D2, affected mother; C1, unaffected father. OD, right eye; OS, left eye. All examinations were unremarkable.

A Chinese family with NSOFCs was recruited for this study, as depicted in Figure 1a. The proband (D1) was a 7-year-old boy who presented with complete left cleft lip and palate, and had undergone previous repair surgeries (Figure 1b). His mother (D2) exhibited bilateral cleft lip and alveolar cleft. The proband’s father (C1) was clinically unaffected. Ophthalmological examinations revealed no definitive corneal abnormalities in the affected family members (Figure A1; Table A2).

To identify candidate variants associated with NSOFCs, WES was carried out on DNA from the three family members. Subsequent analysis revealed a previously unidentified stop-gain mutation located in exon 2 of *TGFBI* (NM_000358.3: c.230C > A), predicted to introduce a premature termination codon (p.Ser77X). Sanger sequencing confirmed the presence of the stop-gain variant. The two affected individuals (D1 and D2) carried the mutation in a heterozygous state, whereas the unaffected member (C1) harbored only wild-type sequences, consistent with an autosomal dominant inheritance pattern in this pedigree (Figure 1c).

The population frequency and annotation of this variant were subsequently evaluated using publicly available genomic databases. This variant has not been documented in any major databases, including the 1000 Genomes Project, ExAC, and gnomAD (exome and genome cohorts) (Table 1). These data suggest that it is either extremely rare or novel in the general population. Collectively, these findings indicate that the heterozygous p.S77X variant represents a novel candidate pathogenic variant associated with NSOFCs in this family.

To further explore the potential contribution of *TGFBI* variation to NSOFCs, a rare heterozygous missense variant, NM_000358.3: c.998C>T (p.Thr333Met), was identified in an unrelated sporadic NSOFC patient and validated by Sanger sequencing. This variant was predicted to have a potentially damaging effect by multiple in silico prediction tools (Figure S1; Table S3), providing additional genetic support for a potential role of *TGFBI* variation in NSOFCs.

### 3.2. Predicting the Pathogenicity of the p.S77X Variant In Silico

**Figure 2:**
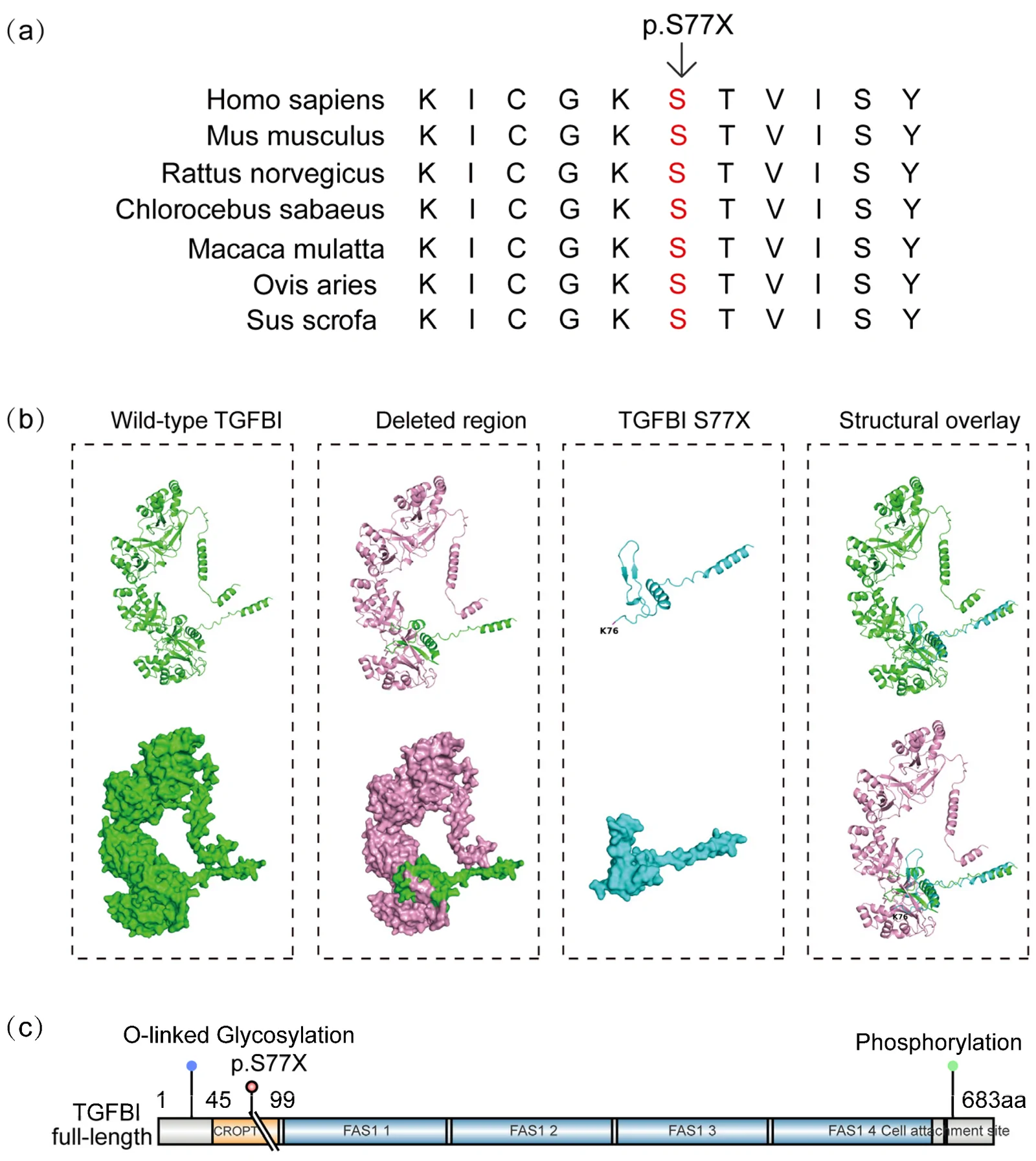
In silico prediction of the p.S77X variant pathogenicity. (**a**) Evolutionary conservation of the *TGFBI* variant site across species. The mutated residue is highlighted in red and indicated by an arrow. TGFBI protein sequences were obtained from https://www.ncbi.nlm.nih.gov/protein/?term=TGFBI, accessed on 16 August 2026. (**b**) Predicted three-dimensional structures of WT TGFBI and p.S77X variant, with a predicted product comprising residues 1–76, together with a superposition of WT and p.S77X variant. Wild-type (green): overall structure. Deleted region (pink): segment absent in the mutant, mapped onto the wild-type structure. Mutant (blue): structure of the truncated protein, superimposed on the wild-type to show structural similarity/differences (structural overlay). Structures were generated using AlphaFold3 and visualized in PyMOL. (**c**) Schematic diagram of the *TGFBI* gene and protein depicting the location of the p.S77X variant relative to functional domains.

Then we assessed the effects of p.S77X on the function of *TGFBI* via in silico analyses. Cross-species multiple sequence alignment demonstrated evolutionary conservation of this residue across all examined species, implicating its potential role in TGFBI protein function (Figure 2a). Moreover, the structure predicted by AlphaFold3 [14] indicated that the p.S77X variant is located within the CROPT region of TGFBI protein and is predicted to produce a protein consisting only of the N-terminal signal peptide (residues 1–23) and a partial fragment of the CROPT domain (residues 45–76) (Figure 2b). This truncation completely abolishes the C-terminal half of the CROPT domain, all four FAS1 domains, and the C-terminal RGD motif (Figure 2c). Such extensive C-terminal loss is predicted to severely disrupt normal TGFBI protein function, which likely contributes to the disease association observed in this non-syndromic orofacial cleft pedigree. In addition, NMDEscPredictor classified the c.230C > A (p.S77X) variant within an NMD-competent region, predicting that the mutant transcript is susceptible to nonsense-mediated mRNA decay (Figure S2). In line with these analytical results, the variant of *TGFBI* was predicted to be deleterious by multiple bioinformatics tools (Table 2). Collectively, these data support the potential pathogenicity of the p.S77X variant.

### 3.3. Localization of TGFBI in the Medial Epithelial Seam in the Fusing Mouse Palate

**Figure 3:**
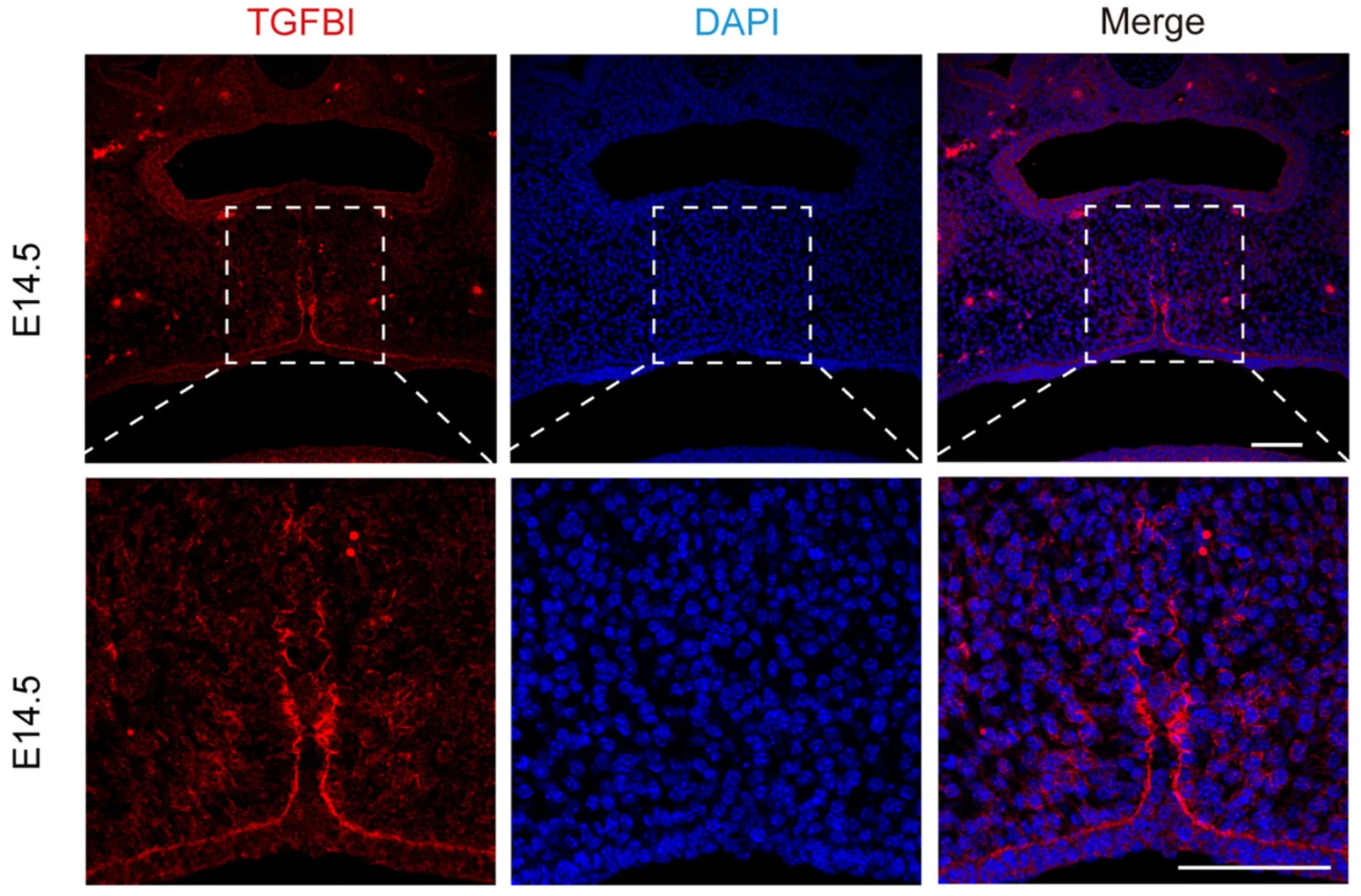
TGFBI expression in the developing mouse palate at embryonic day 14.5 (E14.5). The midline epithelial seam (MES) is outlined by a white dashed box and shown at higher magnification. Scale bars: 100 μm.

**Figure A2:**
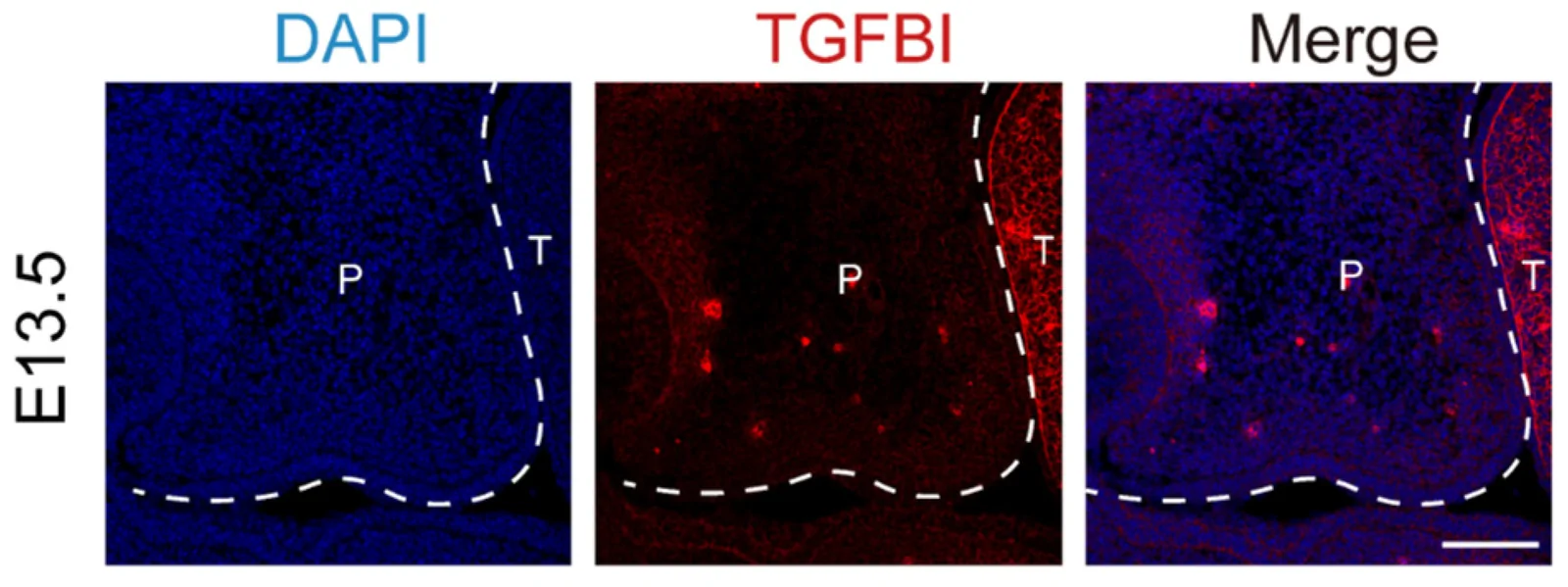
TGFBI expression in the developing mouse palate at embryonic day 13.5 (E13.5). The palate shelf is outlined by a white dashed box. P, palatal shelf; T, tongue. Scale bars: 100 μm.

To explore the mechanistic basis of TGFBI function during palatogenesis, we carried out immunofluorescence (IF) analyses on coronal sections of wild-type mouse embryos isolated at key developmental time points (E14.5). Notably, at E14.5, TGFBI was primarily localized in the MES (Figure 3). To further examine whether TGFBI is constitutively expressed in the palatal epithelium or enriched in the MES upon shelf contact, we assessed TGFBI localization at E13.5 (Figure A2), prior to palatal shelf apposition. At this earlier stage, a low-level TGFBI signal was observed in the palatal epithelium. Upon shelf contact at E14.5, TGFBI protein was detected in the MES (Figure 3 and Figure S3). These findings raise the possibility that TGFBI protein is enriched in the medial edge epithelium following palatal shelf contact, rather than being uniformly distributed throughout the palatal epithelium, and may contribute to palatal shelf fusion during embryonic development.

### 3.4. The p.S77X Mutation Causes Aberrant Protein Expression and Mislocalization

**Figure 4:**
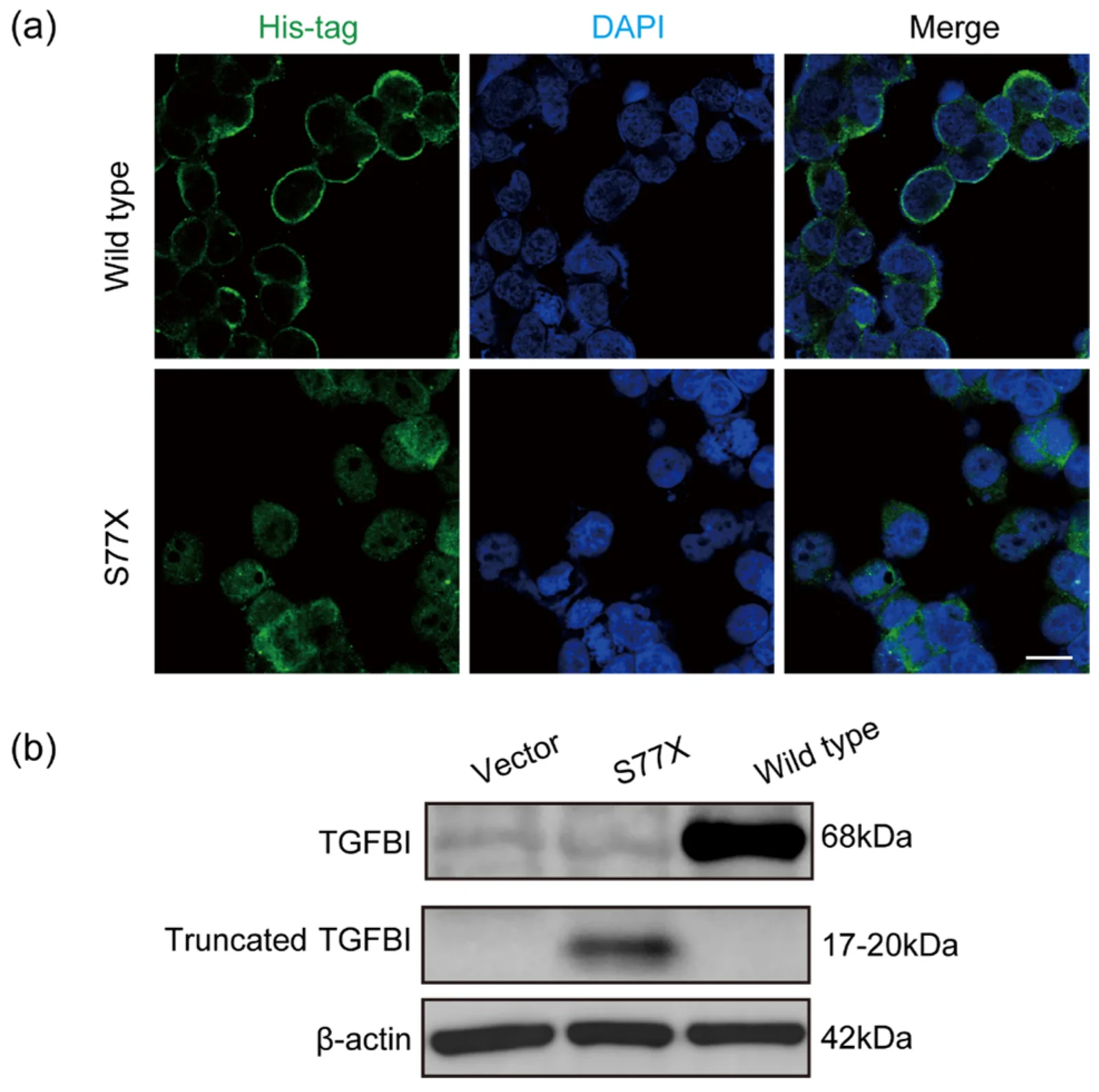
Subcellular localization and expression of TGFBI in transfected HEK293T cells. (**a**) Immunofluorescence staining showing localization of wild-type (WT) and mutant (p.S77X) TGFBI. Cells were harvested 48 h post-transfection. TGFBI was detected using an anti-His tag antibody (green); nuclei were counterstained with DAPI (blue). Scale bar: 100 μm. (**b**) Western blot analysis of TGFBI expression levels in empty vector, wild-type TGFBI, and p.S77X groups. β-actin served as a loading control.

Given its specific expression at the critical site of palate fusion, we sought to determine the molecular and cellular mechanisms underlying the *TGFBI* variant. We generated the His-tagged wild-type, *TGFBI* p.S77X mutant, and empty vector, and then we transfected them into HEK293T cells. Western blot analysis confirmed that wild-type TGFBI was expressed as a band of the expected size (~68 kDa) in vitro. In contrast, cells expressing the p.S77X construct exhibited a lower-molecular-weight product of approximately 17–20 kDa, with no detectable full-length product (Figure 4b). These results demonstrate that the p.S77X variant produces a severely truncated protein, consistent with premature translation termination at amino acid position 77. This truncated product lacks the entire FAS1 domain-containing region and the C-terminal RGD integrin-binding motif, indicating a complete loss of functional domains.

Since TGFBI is a secreted protein, we next examined subcellular localization of TGFBI. The immunofluorescence assay in HEK293T cells revealed that wild-type TGFBI exhibited a localization pattern characteristic of a secreted protein. In contrast, the p.S77X mutant protein was predominantly diffused in the cytoplasm (Figure 4a). These findings indicate that the p.S77X mutation disrupts the normal subcellular localization of TGFBI, which is likely to compromise its biological function.

### 3.5. TGFBI p.S77X Variant Disrupts Apoptosis

**Figure 5:**
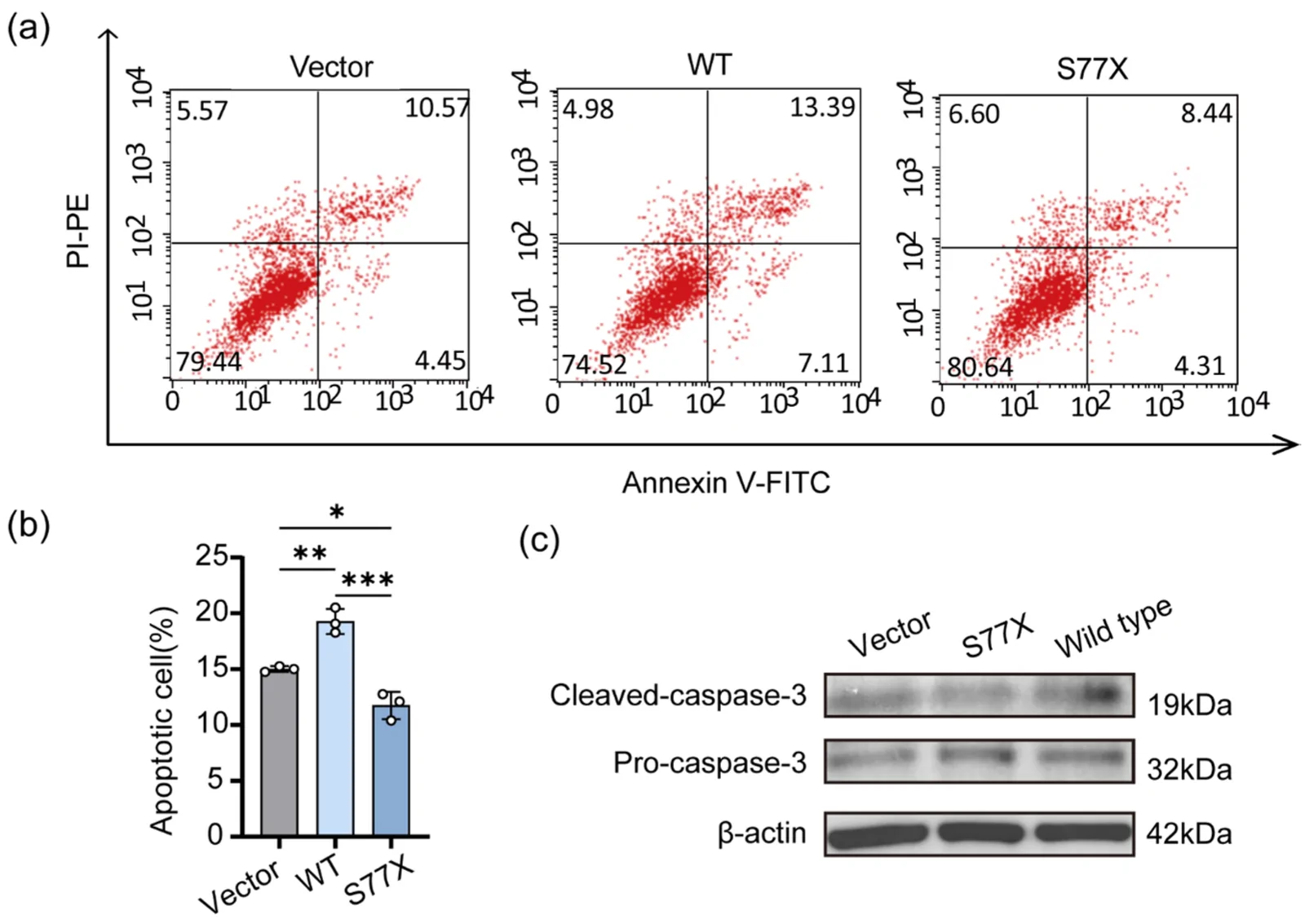
Flow cytometric analysis of apoptosis and Western blot analysis of caspase-3 in transfected HEK293T cells. (**a**,**b**) Representative flow cytometry plots and quantitative analysis of Annexin V-FITC/PI staining in empty vector, wild-type TGFBI (WT), and p.S77X TGFBI(S77X) groups at 48 h post-transfection. Quadrants indicate viable (Annexin V^−^/PI^−^), early apoptotic (Annexin V^+^/PI^−^), late apoptotic (Annexin V^+^/PI^+^), and necrotic (Annexin V^−^/PI^+^) populations. Data are presented as mean ± SD from three independent experiments (n = 3). Statistical significance was determined by one-way ANOVA with Tukey’s multiple comparisons test (* *p* < 0.05; ** *p* < 0.01; *** *p* < 0.001). (**c**) Western blot analysis of caspase-3 and cleaved caspase-3 expression in the three groups at 48 h post-transfection. β-actin served as a loading control.

Previous studies have shown that TGFBI is expressed in the MES during palatal fusion and co-localizes with apoptotic cells [12]. Given that apoptosis participates in periderm degeneration and palatal fusion [23,24], we investigated whether the p.S77X variant affects TGFBI-mediated apoptosis. Flow cytometry analysis revealed that overexpression of wild-type TGFBI potently induced apoptosis in HEK293T cells. As anticipated, cells expressing the p.S77X TGFBI exhibited a significantly reduced apoptotic rate, consistent with the loss of critical functional domains in this variant (Figure 5a,b).

We then examined whether the variant impacted caspase-3 activation, which serves as a central hub in the execution phase of apoptosis. Western blot analysis showed lower cleaved caspase-3 levels in p.S77X-expressing cells than in cells expressing wild-type TGFBI (Figure 5c). These findings suggest that the p.S77X mutation may act as a loss-of-function allele, abolishing the pro-apoptotic activity of *TGFBI*. Given that MES degeneration during palatogenesis involves a precisely regulated apoptotic balance, the loss of pro-apoptotic activity conferred by the p.S77X mutation may disrupt this process, offering a potential mechanistic link between this variant and craniofacial malformations.

**Figure A3:**
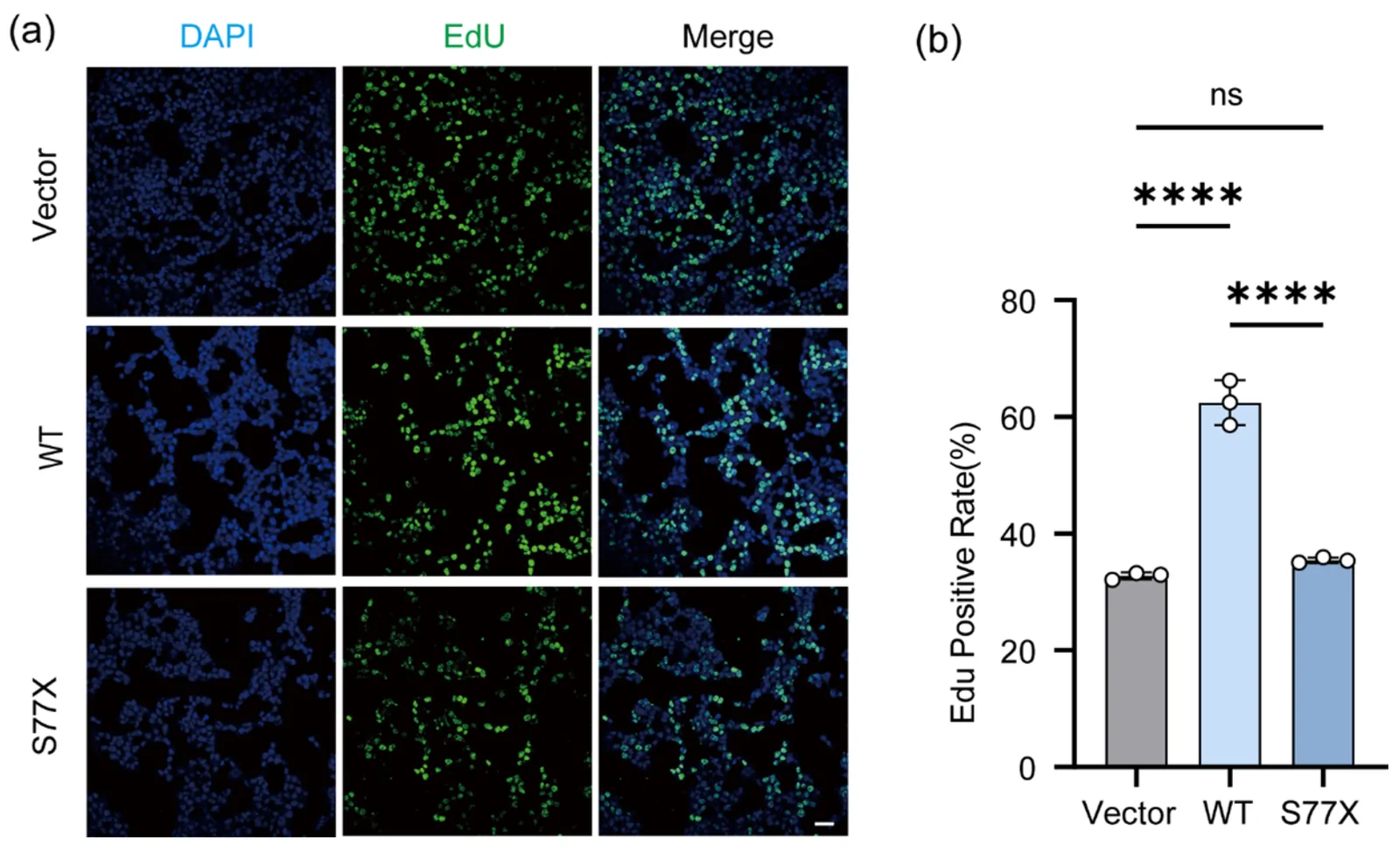
EdU proliferation assay in transfected HEK293T cells. (**a**,**b**) Representative images and quantification of EdU-positive cells (EdU^+^/DAPI^+^) in HEK293T cells transfected with empty vector, WT, or p.S77X plasmids. Scale bars = 100 μm. Data are presented as mean ± SD (n = 3) from three independent experiments. Statistical significance was determined by one-way ANOVA followed by Tukey’s multiple comparison test (**** *p* < 0.0001; ns, not significant).

To further characterize the functional consequences of the p.S77X mutation, we examined its effect on cell proliferation through an EdU incorporation assay. Cells expressing the p.S77X mutant exhibited markedly reduced proliferative capacity compared to those overexpressing wild-type TGFBI, while no significant difference was observed relative to vector-transfected controls (Figure A3). These findings further corroborate the loss-of-function phenotype conferred by the p.S77X mutation.

### 3.6. Dysregulation of the p38-MAPK Signaling Pathway Is Associated with the Apoptotic Defect Caused by the TGFBI p.S77X Variant

**Figure 6:**
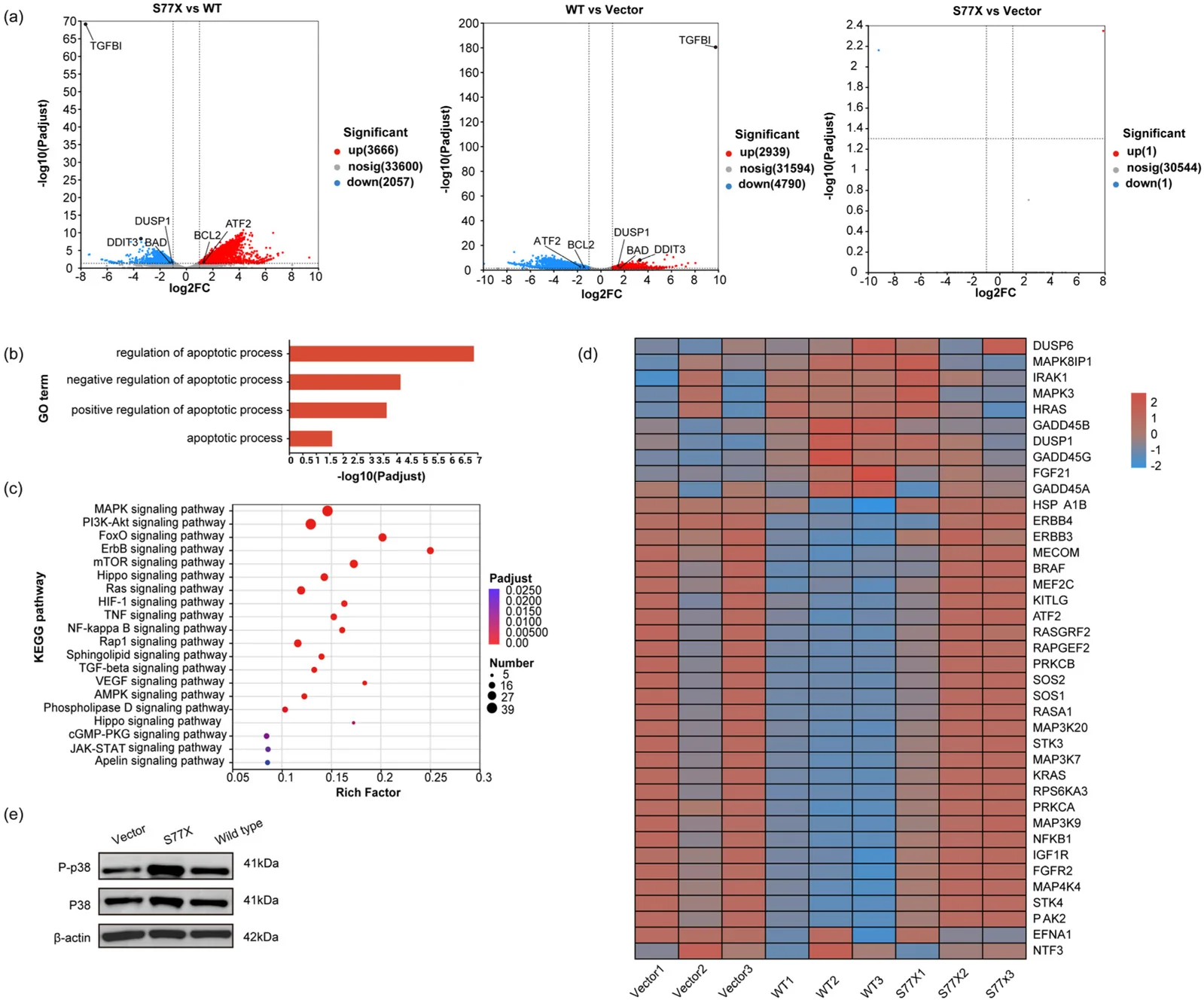
Transcriptomic and Western blot analysis of p38-MAPK signaling. (**a**) Volcano plots of differentially expressed genes (DEGs) among p.S77X, WT *TGFBI*, and vector groups (p.S77X vs. WT, WT vs. vector, and p.S77X vs. vector). Significantly upregulated and downregulated genes (adjusted *p* ≤ 0.05) are shown in red and blue, respectively; non-significant genes are in gray. (**b**) Gene Ontology (GO) enrichment analysis of apoptosis-related terms among DEGs. Enrichment significance is represented as −log10 (adjusted *p*-value). (**c**) KEGG pathway enrichment analysis of apoptosis-related DEGs. Rich factor indicates the degree of enrichment, dot size reflects gene count, and dot color denotes adjusted *p*-value range. (**d**) Heatmap showing MAPK-related gene expression profiles across vector, WT *TGFBI*, and p.S77X samples. Each column represents a sample; each row represents a gene. Expression levels are color-coded from blue (low) to red (high). (**e**) Western blot analysis of total p38 and phosphorylated p38 (p-p38) in empty vector, WT, and p.S77X groups at 48 h post-transfection. β-actin served as a loading control.

**Figure A4:**
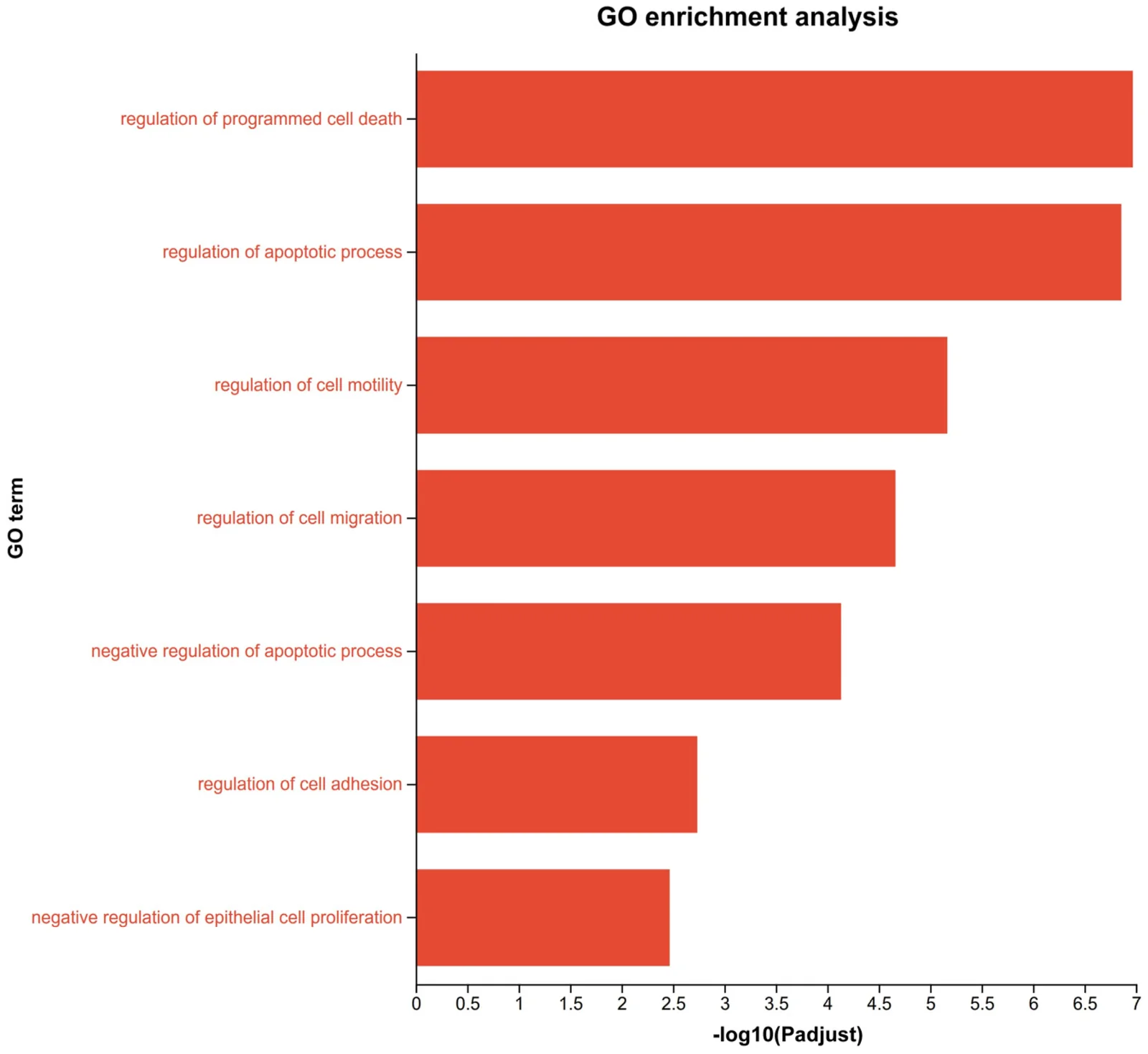
GO enrichment analysis of differentially expressed genes associated with the *TGFBI* p.Ser77X variant. The *x*-axis indicates −log10(adjusted *p*-value), and the *y*-axis represents GO biological process terms.

To explore molecular changes associated with the reduced apoptotic phenotype observed in p.S77X-expressing cells, we performed RNA-seq on the three experimental groups described above. Differential expression analysis revealed that key pro-apoptotic genes, including BCL2-associated agonist of cell death (*BAD*) and DNA damage-inducible transcript 3 (*DDIT3*), were significantly downregulated in cells overexpressing the p.S77X variant compared with those expressing wild-type *TGFBI*. In contrast, the anti-apoptotic regulator *BCL2* was upregulated, consistent with the reduced apoptotic phenotype observed in p.S77X-expressing cells. In addition, activating transcription factor 2 (*ATF2*), a stress-responsive transcription factor associated with MAPK signaling, was upregulated. *TGFBI* itself also showed a pronounced reduction in transcript abundance in p.S77X-expressing cells relative to the wild-type group. Notably, dual specificity phosphatase 1 (*DUSP1*), a negative regulator of MAPK signaling, was also downregulated in the p.S77X group compared with the wild-type group. The WT-versus-vector comparison also showed substantial transcriptional changes, whereas only two genes met the predefined differential-expression criteria in the p.S77X-versus-vector comparison (Figure 6a). RNA-seq revealed enrichment of biological processes related to apoptosis, cell migration, cell adhesion, and epithelial proliferation (Figure A4).

Gene Ontology (GO) enrichment analysis further demonstrated that differentially expressed genes were significantly enriched in biological processes related to apoptosis (Figure 6b). To explore upstream regulatory mechanisms, we performed KEGG pathway enrichment analysis on this apoptosis-associated gene set and identified significant enrichment of the mitogen-activated protein kinase (MAPK) signaling pathway (Figure 6c). Heatmap analysis of MAPK-related genes across the vector, WT *TGFBI*, and p.S77X groups revealed distinct expression patterns among the three experimental conditions (Figure 6d). Notably, *DUSP1* was downregulated in the p.S77X-versus-WT comparison.

Western blot analysis further showed increased phosphorylation of p38 in cells overexpressing the p.S77X variant (Figure 6e). Collectively, these molecular changes are consistent with the reduced apoptotic phenotype observed in p.S77X-expressing cells and suggest potential involvement of p38-MAPK dysregulation in this phenotype.

## 4. Discussion

This study reports a rare heterozygous stop-gain mutation in the *TGFBI* gene segregating with NSOFCs in a human pedigree, providing human genetic evidence supporting a potential contribution of *TGFBI* variation to NSOFC susceptibility. While a previous study suggested that TGFBI (βig-h3) may participate in medial edge epithelium apoptosis during secondary palate fusion [12], no human genetic studies had associated *TGFBI* with NSOFCs prior to this report. To explore the potential molecular mechanism, we performed functional assays in HEK293T cells. The results suggested that the p.S77X mutation impairs both apoptosis and proliferation in HEK293T cells, indicating that this mutation may lead to a loss-of-function phenotype. The observed impairment of apoptosis could be associated with the p38-MAPK signaling pathway and downregulation of key pro-apoptotic genes, such as *BAD* and *DDIT3*. Given that HEK293T cells are not derived from palatal tissue, these findings may provide clues for understanding the role of *TGFBI* in palatal development, but the exact mechanism requires further investigation in palate-relevant cellular or developmental models.

Previous RNA in situ hybridization studies have shown that *Tgfbi* expression is not restricted to epithelial structures during craniofacial development. Schorderet et al. [25] reported embryonic *Tgfbi* expression in the mesenchyme of the first and second branchial arches, and further study [26] showed prominent β-ig mRNA signals in embryonic connective tissues and craniofacial mesenchymal condensations during E12.5–E14.5 mouse development. These transcript-level findings are consistent with the low-level TGFBI protein signals observed in the palatal shelf mesenchyme in our immunofluorescence analysis. Importantly, our results also showed detectable TGFBI protein expression in the MEE before palatal shelf contact, whereas TGFBI protein became more prominently accumulated in the MES at E14.5 after palatal shelf contact. Therefore, TGFBI expression should not be interpreted as being exclusive to the MES; rather, TGFBI appears to be present in both palatal mesenchymal and epithelial compartments, with prominent enrichment in the MES during palatal shelf fusion. Functional analyses indicate that the p.S77X mutation confers a loss-of-function phenotype, as evidenced by impaired pro-apoptotic and proliferative activities. Transcriptomic profiling further revealed that dysregulation of the p38-MAPK signaling pathway correlated with the apoptotic defect observed in cells expressing this variant. Given that p38-MAPK signaling has been implicated as a SMAD-independent mediator downstream of TGF-β/BMP pathways during secondary palate development [27], it is plausible that its dysregulation may contribute to the impaired apoptosis observed in vitro. However, whether apoptosis is strictly required for palatal fusion remains a matter of debate [9,28], and palatal fusion is considered to involve a multi-mechanism cooperative model. Consistent with this view, our study observed altered cell proliferation upon TGFBI p.S77X expression. Notably, further studies examining MES dynamics, epithelial migration, and EMT-related processes will be needed to elucidate the underlying mechanism.

The extracellular matrix protein encoded by *TGFBI* plays diverse roles in maintaining tissue homeostasis [29]. Dysregulated expression of this gene has been implicated in several pathological conditions, including corneal dystrophy [30,31], tumorigenesis and tumor progression [32], pancreatic islet dysfunction [33], metabolic disturbances [34], and impaired skeletal muscle regeneration [35]. Despite this pleiotropy, a genetic association between *TGFBI* and NSOFCs in humans has not been previously established. In this study, we identified rare *TGFBI* variants in NSOFC cases, including a heterozygous truncating variant in a familial case and an additional rare missense variant in an independent sporadic patient. These findings provide additional human genetic evidence supporting a potential broader contribution of *TGFBI* variation to craniofacial development, although further evaluation in larger cohorts and functional studies will be required.

Although *TGFBI* has been canonically linked to corneal dystrophies, ophthalmological examination of the affected family members in our study revealed no signs of corneal abnormalities. This absence of a corneal phenotype is consistent with the established genetic phenomenon whereby different classes of mutations within the same gene can produce divergent disease phenotypes, as exemplified by gain-of-function and loss-of-function variants in *RET*, which are associated with multiple endocrine neoplasia type 2A (MEN2A) and Hirschsprung disease, respectively [36]. In the case of *TGFBI*, the p.S77X mutation identified in our study is a severely truncating variant expected to disrupt normal TGFBI protein function. The molecular consequences of this early truncation appear distinct from those described for many TGFBI-linked corneal dystrophy-associated variants which cause corneal dystrophies through protein aggregation and toxicity. According to a comprehensive review on *TGFBI*-linked corneal dystrophies [31], the disease mechanisms can be divided into two major categories: for granular corneal dystrophy and related types, mutations reduce the solubility of TGFBI, disrupt interactions with the extracellular matrix, and confer resistance to proteolytic degradation, leading to amorphous aggregation of intact or near-intact TGFBI; for lattice corneal dystrophy, mutations (most of which are located in the FAS1-4 domain) may destabilize the protein, rendering it more susceptible to cleavage by the serine protease HtrA1 and releasing amyloidogenic peptides (e.g., the Y571-R588 region), which eventually form amyloid fibrils derived from TGFBI fragments. The same review also lists several nonsense mutations associated with corneal dystrophies (e.g., P130Ter, R179Ter, G470Ter). However, most of these nonsense mutations co-occur with missense mutations that are themselves capable of causing corneal dystrophy through a dominant-negative effect, and these nonsense mutations are located much further downstream in the protein sequence. In contrast, the S77X mutation we identified is located at the very beginning of the CROPT domain, producing an extremely short truncated protein of only 76 amino acids, which lacks all FAS1 domains. Such a short peptide may be rapidly degraded by the cell, and its potential for aggregation or toxicity appears to be low; thus, the underlying mechanism is likely one of loss of function. In addition, in silico analysis predicted that the p.S77X-containing transcript may be susceptible to nonsense-mediated mRNA decay (NMD), although this predicted effect remains to be experimentally validated. However, the endogenous relative abundance of wild-type and mutant *TGFBI* transcripts and proteins could not be determined because patient-derived RNA and protein samples were unavailable. Consistent with a potential loss-of-function interpretation, the first *Tgfbi* knockout mouse model, generated to study the role of TGFBI as a tumor suppressor [37], did not exhibit any obvious ocular phenotype. Furthermore, a later study thoroughly examined the corneal structure of another *Tgfbi*-null mouse model [38] and similarly found no major effect of TGFBI deficiency on overall corneal structure. In fact, partial or complete knockdown of *TGFBI* has been proposed as a potential therapeutic strategy for *TGFBI*-linked corneal dystrophies. These observations are generally consistent with our argument: absence of *TGFBI* may have limited effects on the cornea, whereas corneal disease likely requires the active toxicity of the mutant protein rather than mere loss of function. Therefore, the lack of corneal dystrophy in the affected family members in our study is not entirely unexpected and is compatible with the loss-of-function mechanism we propose.

Studies using constitutive *Tgfbi* knockout (*Tgfbi* −/−) mouse models have reported a range of phenotypes resulting from gene deficiency, including systemic growth retardation [39], increased tumor susceptibility [40], abnormal retinal development [41], islet dysfunction [34,42], and impaired skeletal muscle regeneration [35]. Notably, none of these studies described cleft lip or palate. However, the absence of a reported cleft palate phenotype in constitutive *Tgfbi* knockout mice does not necessarily argue against the involvement of this gene in palatal development. Cleft palate phenotypes in knockout mice often exhibit low penetrance [43]. Even well-established causal genes for orofacial clefts do not always display cleft palate phenotypes with complete penetrance in their knockout models. For instance, Grainyhead-like 3 (*GRHL3*) [44] is widely recognized as a cleft palate gene in both humans and mice, yet *Grhl3* knockout mice exhibit cleft palate with a penetrance of only approximately 17%. Likewise, previous characterization of *Flnb* knockout mice [13] revealed a cleft palate penetrance of approximately 33.3%. These examples illustrate that incomplete penetrance is a common phenomenon in knockout mouse models of orofacial clefting, and that when sample sizes are limited, cleft palate phenotypes may escape detection. Furthermore, the detection of cleft palate requires removal of the mandible to fully expose the palatal shelves, and submucous or partial cleft palate can be easily overlooked in studies not focused on craniofacial development. These considerations suggest that the absence of a documented cleft palate phenotype in *Tgfbi* knockout mice does not definitively exclude a role for TGFBI in palatal fusion. Consistent with a functional requirement, Choi et al. [12] demonstrated that acute antisense oligonucleotide-mediated knockdown of *Tgfbi* at E12.5 resulted in 84% of mice being born with a cleft secondary palate, providing direct in vivo evidence that loss of *TGFBI* function during the critical window of palatal fusion is sufficient to cause cleft palate. Future studies employing additional strains and conditional knockout approaches targeting the palatal epithelium will be valuable to further clarify the relationship between *Tgfbi* loss of function and cleft palate.

## 5. Conclusions

This study identifies a rare heterozygous loss-of-function variant in *TGFBI* associated with NSOFCs. Functional analyses showed impaired apoptosis and altered p38-MAPK signaling in p.S77X-expressing cells. These findings provide evidence supporting a role for *TGFBI* variation in NSOFCs. Further evaluation of *TGFBI* variants in larger cohorts and relevant craniofacial developmental models will help clarify the contribution of *TGFBI* to NSOFC susceptibility and its potential implications for molecular diagnosis and genetic counseling.

## Figures and Tables

**Table 1:** Frequency of *TGFBI* p.S77X variant in public databases.

Variants	Type of Variants	Position Change in Chromosome	cDNA Change	1000 g_ Chinese	1000 g_ EAS	1000 g_ ALL	GnomAD	Genome Cohorts
p.S77X	Stop gain	chr5:135369547C > A	c.230C > A	NR	NR	NR	Not observed (AC = 0; AF = 0)	NR

Note: The version of the chromosome database used in this study is GRCh37/hg19. For querying gnomAD v4.1.1, the corresponding GRCh38/hg38 coordinate, chr5:136033858C > A, was used. The coding sequence position for the *TGFBI* transcript is NM_000358.3. The amino acid position for the *TGFBI* protein is NP_000349.1. NR, not reported; AC, allele count; AF, allele frequency.

**Table 2:** Pathogenicity of *TGFBI* p.S77X variant.

Variants	MutationTaster		CADD		LRT	
	Score	Prediction	Score	Prediction	Score	Prediction
p.S77X	1	Disease causing automatic	37	Highly likely deleterious	0.000	Deleterious

## Data Availability

The raw data have been deposited in the NCBI Sequence Read Archive (SRA) under BioProject accession number PRJNA1434630.

## Supplementary Materials

The following supporting information can be downloaded at: https://www.mdpi.com/article/10.3390/genes17090999/s1, Figure S1: Sanger sequencing chromatograms of a sporadic NSOFC patient; Figure S2: Prediction of NMD for the *TGFBI* c.230C > A (p.S77X) variant; Figure S3: TGFBI expression in the developing mouse palate at embryonic day 14.5 (E14.5); Table S1: One hundred and forty-three candidate genes; Table S2: Twenty-eight candidate genes; Table S3: Annotation of the rare *TGFBI* missense variant identified in a sporadic NSOFC patient.

## Abbreviations

The following abbreviations are used in this manuscript:

NSOFCs	Non-syndromic orofacial clefts
WES	Whole-exome sequencing
*TGFBI*	Transforming growth factor beta-induced
MAPK	Mitogen-activated protein kinase
CL	Cleft lip
CP	Cleft palate
CLP	Cleft lip and palate
GWAS	Genome-wide association study
*IRF6*	Interferon regulatory factor 6
*GRHL3*	Grainyhead-like transcription factor 3
βig-H3	Transforming growth factor-β-induced gene clone 3
MEE	Medial edge epithelium
MES	Medial epithelial seam
RGD	Arginine–Glycine–Aspartic acid
TGFβ	Transforming growth factor-beta
E	Embryonic day
CROPT	Cysteine-rich EMI domain-containing protein
FAS1	Fasciclin 1 domain
HEK293T	Human embryonic kidney 293T cells
His	Histidine tag
WT	Wild-type
Bulk RNA-seq	Bulk RNA sequencing
KEGG	Kyoto Encyclopedia of Genes and Genomes
GO	Gene Ontology
BMP	Bone morphogenetic protein
MEPE Cells	Mouse Embryonic Palatal Epithelial Cells
*BAD*	BCL2-associated agonist of cell death
*DDIT3*	DNA damage-inducible transcript 3
*ATF2*	Activating transcription factor 2
*DUSP1*	Dual specificity phosphatase 1
MEN2A	Multiple endocrine neoplasia type 2A

## Appendix A

**Table A1:** Final-stage candidate variants after stepwise filtering and rationale for prioritization.

Gene	Variant	Rationale
*TGFBI*	c.230C > A p.S77X	Selected: downstream effector of TGF-β3 signaling; Choi et al. [12] showed *Tgfbi* knockdown causes cleft palate in 84% of mice
*TTN*	c.35730delA p.G11911Afs*59	Excluded: identical variant reported in congenital titinopathy patient without cleft palate [45]
*TSPAN12*	c.550dupA p.R184Kfs*17	Excluded: no reported association with orofacial clefting
*RMND5B*	c.1A > G	Excluded: no reported association with orofacial clefting

Note: The version of the chromosome database used in this study is GRCh37/hg19. The coding sequence position for the *TGFBI* transcript is NM_000358.3. The coding sequence position for the *TTN* transcript is NM_001267550.2. The coding sequence position for the *TSPAN12* transcript is NM_012338.4. The coding sequence position for the *RMND5B* transcript is NM_022762.5. The asterisk (*) denotes a translation termination codon in protein-level variant nomenclature.

**Table A2:** Slit-lamp examination of the pedigree members.

Parameter	D1	D2	C1
Eyelids	Normal	Normal	Normal
Conjunctiva	No congestion	No congestion	No congestion
Cornea	Transparent	Transparent	Transparent
Anterior chamber	Deep and clear	Deep and clear	Deep and clear
Pupil	Normal	Normal	Normal
Lens	Transparent	Transparent	Transparent

Note: Slit-lamp examination was performed on all three family members. The proband (D1) and the affected mother (D2), both carrying the *TGFBI* c.230C > A (p.S77X) mutation, showed no signs of corneal opacity or other ocular abnormalities characteristic of *TGFBI*-related corneal dystrophies. Clinical slit-lamp photographs were not available; the examination findings were documented in written clinical records.
