# Supplementary material for: A Novel Loss-of-Function Variant in TGFBI Associated with Non-Syndromic Orofacial Clefts in a Chinese Pedigree
Source: Genes (Basel). 2026 Aug 25;17(9):999. doi: 10.3390/genes17090999 (PMC13606577; doi:10.3390/genes17090999)
Supplement: Supplementary file 1 [file genes-17-00999-s001.zip › Supplemental Figures legend.pdf]

Supplemental Figure

Figure S1. Sanger sequencing chromatograms of a sporadic NSOFC patient
Solid boxes denote substituted nucleotides. wt, wild-type allele; mut, mutant allele.

Figure S2. Prediction of NMD for the *TGFBI* c.230C>A (p.S77X) variant. The p.S77X variant (blue) is located within an NMD+ region (red), indicating that the mutant transcript is predicted to be susceptible to nonsense-mediated mRNA decay.

Figure S3. TGFBI expression in the developing mouse palate at embryonic day 14.5 (E14.5). The midline epithelial seam (MES) is outlined by a white dashed box. Scale bars: 50 μm.
