# Supplementary material for: A Novel Loss-of-Function Variant in TGFBI Associated with Non-Syndromic Orofacial Clefts in a Chinese Pedigree
Source: Genes (Basel). 2026 Aug 25;17(9):999. doi: 10.3390/genes17090999 (PMC13606577; doi:10.3390/genes17090999)
Supplement: Supplementary file 1 [file genes-17-00999-s001.zip › Table S3.pdf]

Table S3. Annotation of the rare TGFBI missense variant identified in a sporadic NSOFCs patient.

Gene	Type	Het/ Hom	Position change in chromosome	cDNA change	Amino acid substitution	Information in databases			Predicted pathogenicity	
						dbSNP	1000G (All)	1000G (EAS)	SIFT	PolyPhen-2
TGFBI	missense	Het	chr5:135388680C>T	c.998C>T	p.T333M	rs767052038	NR	NR	0.002(D)	0.982,0.524 (D,P)

[1] The version of chromosome database used in this study is GRCh37/hg19.

[2] "NR": Not reported.

[3] "Het": Heterozygous; "Hom": Homozygous.

[4] "D": Damaging; "P": Possibly damaging.
